# *N*,*N*′-Bis(4-bromo-2-fluoro­benzyl­idene)ethane-1,2-diamine

**DOI:** 10.1107/S1600536808029000

**Published:** 2008-09-13

**Authors:** Hoong-Kun Fun, Reza Kia

**Affiliations:** a X-ray Crystallography Unit, School of Physics, Universiti Sains Malaysia, 11800 USM, Penang, Malaysia

## Abstract

The mol­ecule of the title Schiff base compound, C_16_H_12_Br_2_F_2_N_2_, lies across a crystallographic inversion centre and adopts an *E* configuration with respect to the azomethine C=N bonds. The imino groups are coplanar with the aromatic rings. Within the mol­ecule, the planar units are parallel, but extend in opposite directions from the dimethyl­ene bridge. An inter­esting feature of the crystal structure is the short inter­molecular Br⋯F inter­actions [3.2347 (16) Å, which is shorter than the sum of the van der Waals radii of these atoms]. These inter­actions link neighbouring mol­ecules along the *c *axis. The crystal structure is further stabilized by inter­molecular C—H⋯N hydrogen bonds.

## Related literature

For bond-length data, see: Allen *et al.* (1987 ▶). For halogen–halogen inter­actions, see: Ramasubbu *et al.* (1986 ▶); Brammer *et al.* (2003 ▶). For related structures, see, for example: Fun & Kia (2008*a* ▶,*b* ▶,*c* ▶): Fun *et al.* (2008 ▶). For Schiff base complexes and their applications, see, for example: Pal *et al.* (2005 ▶); Calligaris & Randaccio, (1987 ▶); Hou *et al.* (2001 ▶); Ren *et al.* (2002 ▶).
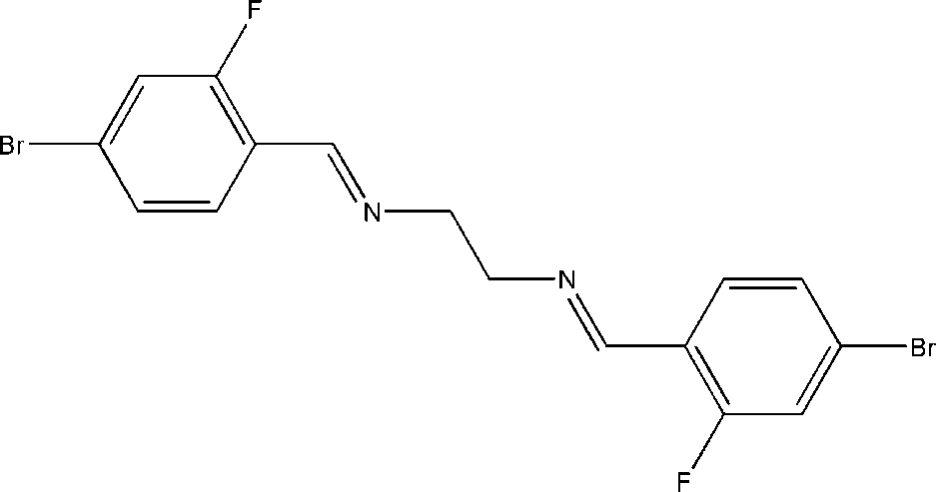


## Experimental

### 

#### Crystal data

C_16_H_12_Br_2_F_2_N_2_
*M* *_r_* = 430.10
Monoclinic, 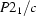

*a* = 4.1981 (1) Å
*b* = 14.6190 (3) Å
*c* = 12.8861 (3) Å
β = 104.751 (2)°
*V* = 764.78 (3) Å^3^
*Z* = 2
Mo *K*α radiation
μ = 5.32 mm^−1^
*T* = 100.0 (1) K
0.51 × 0.07 × 0.05 mm

#### Data collection

Bruker SMART APEXII CCD area-detector diffractometer
Absorption correction: multi-scan (*SADABS*; Bruker, 2005 ▶) *T*_min_ = 0.172, *T*_max_ = 0.769
19361 measured reflections
2631 independent reflections
1907 reflections with *I* > 2σ(*I*)
*R*_int_ = 0.050

#### Refinement

*R*[*F* ^2^ > 2σ(*F* ^2^)] = 0.040
*wR*(*F* ^2^) = 0.100
*S* = 1.05
2631 reflections
100 parameters
H-atom parameters constrained
Δρ_max_ = 1.40 e Å^−3^
Δρ_min_ = −0.85 e Å^−3^

### 

Data collection: *APEX2* (Bruker, 2005 ▶); cell refinement: *APEX2*; data reduction: *SAINT* (Bruker, 2005 ▶); program(s) used to solve structure: *SHELXTL* (Sheldrick, 2008 ▶); program(s) used to refine structure: *SHELXTL*; molecular graphics: *SHELXTL*; software used to prepare material for publication: *SHELXTL* and *PLATON* (Spek, 2003 ▶).

## Supplementary Material

Crystal structure: contains datablocks global, I. DOI: 10.1107/S1600536808029000/at2629sup1.cif

Structure factors: contains datablocks I. DOI: 10.1107/S1600536808029000/at2629Isup2.hkl

Additional supplementary materials:  crystallographic information; 3D view; checkCIF report

## Figures and Tables

**Table 1:** Hydrogen-bond geometry (Å, °)

*D*—H⋯*A*	*D*—H	H⋯*A*	*D*⋯*A*	*D*—H⋯*A*
C2—H2*A*⋯N1^i^	0.93	2.53	3.386 (3)	154

Symmetry code: (i) 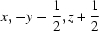.

**Fig. 1.**
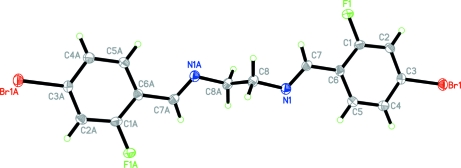
The molecular structure of (I) with atom labels and 50% probability ellipsoids for non-H atoms [symmetry code for A: -x, -y, -z.

**Fig. 2.**
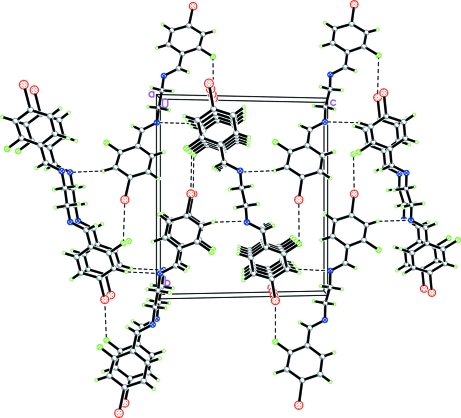
The crystal packing of (I), viewed down the a-axis, shows linking of molecules by Br···F contacts along the c-axis and the stacking of these molecules down the a-axis. Intermolecular interactions are shown as dashed lines.

## supplementary crystallographic information

### Comment

Schiff bases are one of most prevalent mixed-donor ligands in the field of coordination chemistry. There has been growing interest in Schiff base ligands, mainly because of their wide application in the field of biochemistry, synthesis, and catalysis (Pal *et al.*, 2005; Hou *et al.*, 2001; Ren *et al.*, 2002). Many Schiff base complexes have been structurally characterized, but only a relatively small number of free Schiff bases have been characterized (Calligaris & Randaccio, 1987). As an extension of our work (Fun & Kia 2008*a,b,c*; Fun *et al.*,  2008) on the structural characterization of Schiff base ligands, and the halogen-halogen interactions in the halogen-subtituated Schiff bases, the title compound (I), is reported here.

The molecule of the title compound, (I), (Fig. 1), lies across a crystallographic inversion centre and adopts an *E* configuration with respect to the azomethine C═N bond. The bond lengths (Allen *et al.*, 1987) and angles are within normal ranges and are comparable with the related structures (Fun & Kia 2008*a,b,c*; Fun *et al.*,  2008). The two planar units are parallel but extend in opposite directions from the dimethylene bridge. The interesting feature of the crystal structure is the short intermolecular Br···F interactions [symmetry code: 1 - *x*, -1/2 + *y*, 1/2 - *z*] with the distance of 3.2347 (16) Å, which is shorter than the sum of the van der Waals radii of these atoms. The directionality of these interactions, C—*X*···*X*—C (*X* = halogens), has been attributed to anisotropic van der Waals radii for terminally bound halogens or ascribed to donor-acceptor interactions involving a lone pair donor orbital on one halogen and a C—*X*σ^*^ acceptor orbital on the other (Ramasubbu *et al.*, 1986; Brammer *et al.*, 2003). These interactions link neighbouring molecules along the *c*-axis (Fig. 2). The crystal structure is further stabilized by intermolecular C—H···N hydrogen bonds (Table 1).

### Experimental

The synthetic method has been described earlier (Fun & Kia 2008*a*). Single crystals suitable for *X*-ray diffraction were obtained by evaporation of an ethanol solution at room temperature.

### Refinement

All of the hydrogen atoms were positioned geometrically with C—H = 0.93 or 0.97 Å and refined in riding model with *U*_iso_ (H) = 1.2 *U*_eq_ (C). The highest peak is located 1.73 Å from Br1 and the deepest hole is located 0.7 Å from Br1.

### Crystal data

**Table:**

C_16_H_12_Br_2_F_2_N_2_	*F*(000) = 420
*M**_r_* = 430.10	*D*_x_ = 1.868 Mg m^−^^3^
Monoclinic, *P*2_1_/*c*	Mo *K*α radiation, λ = 0.71073 Å
Hall symbol: -P 2ybc	Cell parameters from 5643 reflections
*a* = 4.1981 (1) Å	θ = 3.2–30.0°
*b* = 14.6190 (3) Å	µ = 5.32 mm^−^^1^
*c* = 12.8861 (3) Å	*T* = 100 K
β = 104.751 (2)°	Needle, colourless
*V* = 764.78 (3) Å^3^	0.51 × 0.07 × 0.05 mm
*Z* = 2	

### Data collection

**Table:**

Bruker SMART APEXII CCD area-detector diffractometer	2631 independent reflections
Radiation source: fine-focus sealed tube	1907 reflections with *I* > 2σ(*I*)
graphite	*R*_int_ = 0.050
φ and ω scans	θ_max_ = 32.0°, θ_min_ = 2.2°
Absorption correction: multi-scan (SADABS; Bruker, 2005)	*h* = −6→6
*T*_min_ = 0.172, *T*_max_ = 0.770	*k* = −21→21
19361 measured reflections	*l* = −19→18

### Refinement

**Table:**

Refinement on *F*^2^	Primary atom site location: structure-invariant direct methods
Least-squares matrix: full	Secondary atom site location: difference Fourier map
*R*[*F*^2^ > 2σ(*F*^2^)] = 0.040	Hydrogen site location: inferred from neighbouring sites
*wR*(*F*^2^) = 0.100	H-atom parameters constrained
*S* = 1.05	*w* = 1/[σ^2^(*F*_o_^2^) + (0.0539*P*)^2^] where *P* = (*F*_o_^2^ + 2*F*_c_^2^)/3
2631 reflections	(Δ/σ)_max_ < 0.001
100 parameters	Δρ_max_ = 1.40 e Å^−^^3^
0 restraints	Δρ_min_ = −0.86 e Å^−^^3^

### Special details

**Table:**

Geometry. All e.s.d.'s (except the e.s.d. in the dihedral angle between two l.s. planes) are estimated using the full covariance matrix. The cell e.s.d.'s are taken into account individually in the estimation of e.s.d.'s in distances, angles and torsion angles; correlations between e.s.d.'s in cell parameters are only used when they are defined by crystal symmetry. An approximate (isotropic) treatment of cell e.s.d.'s is used for estimating e.s.d.'s involving l.s. planes.
Refinement. Refinement of *F*^2^ against ALL reflections. The weighted *R*-factor *wR* and goodness of fit *S* are based on *F*^2^, conventional *R*-factors *R* are based on *F*, with *F* set to zero for negative *F*^2^. The threshold expression of *F*^2^ > σ(*F*^2^) is used only for calculating *R*-factors(gt) *etc*. and is not relevant to the choice of reflections for refinement. *R*-factors based on *F*^2^ are statistically about twice as large as those based on *F*, and *R*- factors based on ALL data will be even larger.

### Fractional atomic coordinates and isotropic or equivalent isotropic displacement parameters (Å^2^)

**Table:**

	*x*	*y*	*z*	*U*_iso_*/*U*_eq_	
Br1	−0.74079 (7)	−0.51485 (2)	0.18481 (2)	0.02329 (11)	
F1	−0.0347 (4)	−0.22639 (11)	0.31348 (12)	0.0267 (4)	
N1	−0.0960 (6)	−0.12204 (15)	0.01873 (19)	0.0192 (5)	
C1	−0.2112 (7)	−0.27577 (19)	0.2280 (2)	0.0199 (5)	
C2	−0.3675 (7)	−0.35336 (19)	0.2493 (2)	0.0206 (6)	
H2A	−0.3576	−0.3716	0.3192	0.025*	
C3	−0.5404 (7)	−0.40314 (18)	0.1619 (2)	0.0181 (5)	
C4	−0.5632 (7)	−0.37558 (19)	0.0569 (2)	0.0215 (6)	
H4A	−0.6819	−0.4099	−0.0009	0.026*	
C5	−0.4047 (7)	−0.29567 (19)	0.0405 (2)	0.0208 (6)	
H5A	−0.4212	−0.2761	−0.0293	0.025*	
C6	−0.2210 (6)	−0.24370 (18)	0.1260 (2)	0.0174 (5)	
C7	−0.0381 (6)	−0.16150 (18)	0.1090 (2)	0.0182 (5)	
H7A	0.1239	−0.1380	0.1659	0.022*	
C8	0.1076 (7)	−0.04254 (19)	0.0114 (2)	0.0208 (5)	
H8A	0.2171	−0.0516	−0.0456	0.025*	
H8B	0.2750	−0.0349	0.0783	0.025*	

### Atomic displacement parameters (Å^2^)

**Table:**

	*U*^11^	*U*^22^	*U*^33^	*U*^12^	*U*^13^	*U*^23^
Br1	0.02442 (16)	0.01848 (15)	0.02622 (17)	−0.00289 (11)	0.00507 (11)	0.00316 (11)
F1	0.0383 (10)	0.0214 (9)	0.0173 (8)	−0.0032 (7)	0.0014 (7)	−0.0029 (6)
N1	0.0213 (11)	0.0165 (11)	0.0209 (11)	−0.0001 (9)	0.0072 (9)	0.0004 (9)
C1	0.0220 (13)	0.0195 (14)	0.0163 (12)	0.0024 (10)	0.0015 (10)	−0.0035 (10)
C2	0.0261 (14)	0.0187 (13)	0.0168 (13)	0.0035 (10)	0.0051 (11)	0.0018 (10)
C3	0.0191 (12)	0.0153 (12)	0.0209 (13)	−0.0006 (9)	0.0066 (10)	0.0012 (10)
C4	0.0221 (13)	0.0231 (14)	0.0187 (13)	−0.0001 (11)	0.0042 (11)	−0.0006 (11)
C5	0.0231 (13)	0.0197 (13)	0.0185 (13)	0.0003 (10)	0.0034 (11)	0.0022 (10)
C6	0.0192 (13)	0.0142 (12)	0.0196 (13)	0.0031 (9)	0.0064 (10)	0.0015 (10)
C7	0.0182 (12)	0.0151 (12)	0.0210 (13)	0.0016 (9)	0.0042 (10)	−0.0022 (10)
C8	0.0178 (12)	0.0194 (12)	0.0251 (14)	−0.0027 (10)	0.0055 (10)	−0.0026 (11)

### Geometric parameters (Å, °)

**Table:**

Br1—C3	1.895 (3)	C4—C5	1.387 (4)
F1—C1	1.366 (3)	C4—H4A	0.9300
N1—C7	1.265 (3)	C5—C6	1.398 (4)
N1—C8	1.460 (4)	C5—H5A	0.9300
C1—C2	1.373 (4)	C6—C7	1.472 (4)
C1—C6	1.387 (4)	C7—H7A	0.9300
C2—C3	1.382 (4)	C8—C8^i^	1.521 (6)
C2—H2A	0.9300	C8—H8A	0.9700
C3—C4	1.391 (4)	C8—H8B	0.9700
			
C7—N1—C8	116.3 (2)	C4—C5—H5A	119.1
F1—C1—C2	117.7 (2)	C6—C5—H5A	119.1
F1—C1—C6	117.7 (2)	C1—C6—C5	116.2 (2)
C2—C1—C6	124.6 (3)	C1—C6—C7	121.8 (2)
C1—C2—C3	116.8 (3)	C5—C6—C7	122.0 (2)
C1—C2—H2A	121.6	N1—C7—C6	121.6 (2)
C3—C2—H2A	121.6	N1—C7—H7A	119.2
C2—C3—C4	122.2 (2)	C6—C7—H7A	119.2
C2—C3—Br1	119.2 (2)	N1—C8—C8^i^	109.6 (3)
C4—C3—Br1	118.6 (2)	N1—C8—H8A	109.8
C5—C4—C3	118.4 (3)	C8^i^—C8—H8A	109.8
C5—C4—H4A	120.8	N1—C8—H8B	109.8
C3—C4—H4A	120.8	C8^i^—C8—H8B	109.8
C4—C5—C6	121.9 (3)	H8A—C8—H8B	108.2
			
F1—C1—C2—C3	−179.0 (2)	F1—C1—C6—C7	2.4 (4)
C6—C1—C2—C3	1.3 (4)	C2—C1—C6—C7	−177.9 (3)
C1—C2—C3—C4	−1.5 (4)	C4—C5—C6—C1	−1.1 (4)
C1—C2—C3—Br1	176.3 (2)	C4—C5—C6—C7	176.7 (3)
C2—C3—C4—C5	0.4 (4)	C8—N1—C7—C6	−178.5 (2)
Br1—C3—C4—C5	−177.4 (2)	C1—C6—C7—N1	−165.9 (3)
C3—C4—C5—C6	1.0 (4)	C5—C6—C7—N1	16.4 (4)
F1—C1—C6—C5	−179.8 (2)	C7—N1—C8—C8^i^	−115.8 (3)
C2—C1—C6—C5	−0.1 (4)		

Symmetry codes: (i) −*x*, −*y*, −*z*.

### Hydrogen-bond geometry (Å, °)

**Table:**

*D*—H···*A*	*D*—H	H···*A*	*D*···*A*	*D*—H···*A*
C2—H2A···N1^ii^	0.93	2.53	3.386 (3)	154.

Symmetry codes: (ii) *x*, −*y*−1/2, *z*+1/2.
